# Confronting the vector leptoquark hypothesis with new low- and high-energy data

**DOI:** 10.1140/epjc/s10052-023-11304-5

**Published:** 2023-02-16

**Authors:** Jason Aebischer, Gino Isidori, Marko Pesut, Ben A. Stefanek, Felix Wilsch

**Affiliations:** Physik-Institut, Universität Zürich, 8057 Zürich, Switzerland

## Abstract

In light of new data we present an updated phenomenological analysis of the simplified $U_1$-leptoquark model addressing charged-current *B*-meson anomalies. The analysis shows a good compatibility of low-energy data (dominated by the lepton flavor universality ratios $R_D$ and $R_{D^*}$) with the high-energy constraints posed by $pp\rightarrow \tau {\bar{\tau }}$ Drell-Yan data. We also show that present data are well compatible with a framework where the leptoquark couples with similar strength to both left- and right-handed third-generation fermions, a scenario that is well-motivated from a model building perspective. We find that the high-energy implications of this setup will be probed at the 95% confidence level in the high-luminosity phase of the LHC.

## Introduction

The hypothesis of a vector leptoquark field ($U_1$), transforming as $({\textbf{3}},{\textbf{1}},2/3)$ under the Standard Model (SM) gauge symmetry, with a mass in the TeV range has attracted intense interest in the last few years. At first, this interest arose from a purely phenomenological perspective, when it was realized that this field could offer a combined explanation of both the charged- and neutral-current *B*-meson anomalies [1–4]. In fact, it was soon realized that the $U_1$ hypothesis is the only single-mediator explanation of the two sets of anomalies, while remaining well compatible with all available data [5–7]. After these phenomenological analyses, a purely theoretical interest also began to grow with the realization that the $U_1$ hypothesis naturally points to an underlying *SU*(4) Pati-Salam like [8] symmetry unifying quarks and leptons [3]. In addition, the flavor structure of the $U_1$ couplings suggested by data hinted towards new dynamics potentially connected to the origin of the Yukawa hierarchies [3, 5].

These observations motivated an intense theoretical effort to build more complete models hosting a TeV-scale $U_1$ field. Among them, a particularly compelling class is that of so-called “4321” gauge models [9–14]. In these models, the SM gauge symmetry is extended to $SU(4)_h\times SU(3)_l \times SU(2)_L \times U(1)_X$ [9], allowing the SM fermions to have flavor non-universal gauge charges [10], such that the $U_1$ is coupled mainly to the heavy third-generation fermions. It has also been proposed that the 4321 structure at the TeV scale, whose phenomenology has been analysed in detail in [15, 16], could be the first layer of a more ambitious multi-scale construction [10, 17–19]. This class of models is able to explain both the origin of the Yukawa hierarchies as well as stabilize the SM Higgs sector, as in [20–22]. Alternative approaches to embed the $U_1$ in extended gauge groups and/or describe it in the context of composite models have been proposed in [23–28, 28–30], while additional recent phenomenological studies about the $U_1$ have been presented in [31–34].

Since the latest phenomenological studies, two sets of experimental data providing additional information about the leading $U_1$ couplings to third-generation fermions have appeared. On the low energy side, LHCb has reported an updated measurement of the Lepton Flavor Universality (LFU) ratio $R_{D^*}$ and the first measurement of $R_D$ at a hadron collider [35], with the ratios defined as

1 $$\begin{aligned} R_H = \Gamma (B \rightarrow H \tau {\bar{\nu }})/\Gamma (B \rightarrow H \mu {\bar{\nu }})\,. \end{aligned}$$

On the high-energy side, new bounds on non-standard contributions to $\sigma (pp \rightarrow \tau {\bar{\tau }})$ have been reported by CMS [36, 37]. As pointed out first in [38], the $pp \rightarrow \tau {\bar{\tau }}$ process via *t*-channel $U_1$ exchange is a very sensitive probe of the $U_1$ couplings to third-generation fermions, even for relatively high $U_1$ masses. Interestingly enough, CMS data currently indicates a $3\sigma$ excess of events in $pp \rightarrow \tau {\bar{\tau }}$, well compatible with a possible $U_1$ contribution [37]. However, no excess in $pp \rightarrow \tau {\bar{\tau }}$ is observed by ATLAS [39] (although this analysis is not optimized for non-resonant $U_1$ contributions), making drawing any conclusions about this excess premature. Still, these new data motivate a closer investigation about the compatibility of low- and high-energy observables under the $U_1$ hypothesis, which is the main goal of this paper. We will pursue this goal in a general, bottom-up perspective by focusing only on the leading $U_1$ couplings to third-generation leptons while avoiding details that depend on the specific ultraviolet (UV) completions of the model as much as possible.

The paper is organized as follows: in Sect. 2 we introduce the simplified model employed to analyze both low- and high-energy data. Particular attention is devoted to determine the (quark) flavor structure of the $U_1$ couplings, which is essential to relate the different amplitudes we are interested in ($b\rightarrow c\tau {\bar{\nu }}$ and $b\rightarrow u\tau {\bar{\nu }}$ at low energies, $b {\bar{b}} \rightarrow \tau {\bar{\tau }}$ at high energy). In Sect. 3, we perform a $\chi ^2$-fit in our simplified model to determine the parameter space preferred by low-energy data. We then investigate the compatibility of the preferred low-energy parameter space with high-$p_T$ constraints from $pp \rightarrow \tau {\bar{\tau }}$. The conclusions are summarised in Sect. 4. The Appendix A contains a summary of the preferred parameter-space region in view of future searches.

## Model

The starting point of our analysis is the hypothesis of a massive $U_1$ field, coupled dominantly to third-generation fermions. Focusing on third-generation leptons, and assuming no leptoquark (LQ) couplings to light right-handed fields (which are severely constrained by data, see e.g. [15, 40]), we restrict our attention to the following terms in the LQ current:

2 $$\begin{aligned} J_U^\mu =\frac{g_U}{ \sqrt{2} } \left[ {\overline{q}}^3_L\gamma ^\mu \ell ^3_L+\beta _R\,{\overline{d}}^3_R\gamma ^\mu e^3_R+ \sum _{k=1,2} \epsilon _{q_k} \, {\overline{q}}^k_L\gamma ^\mu \ell ^3_L\right] \,. \end{aligned}$$

Here the right-handed fields and the lepton doublet are understood to be in the corresponding mass-eigenstate basis, while the basis for the left-handed quarks is left generic and will be discussed in detail later on.

Integrating out the LQ field at the tree level leads to the effective interactions

3 $$\begin{aligned} {{\mathcal {L}}}^\textrm{LQ}_\textrm{EFT}= & {} -\frac{2}{v^2} \Big [C_{LL}^{ij\alpha \beta }\, {{\mathcal {O}}}^{ij\alpha \beta }_{LL} + C_{RR}^{ij\alpha \beta }\, {{\mathcal {O}}}^{ij\alpha \beta }_{RR} \nonumber \\{} & {} + \left( C_{LR}^{ij\alpha \beta }\, {{\mathcal {O}}}^{ij\alpha \beta }_{LR}+\mathrm{h.c.} \right) \Big ], \end{aligned}$$

where

$$\begin{aligned} {{\mathcal {O}}}^{ij\alpha \beta }_{LL}&= ({\bar{q}}_{L}^{\,i} \gamma _{\mu } \ell _{L}^{\alpha }) ({\bar{\ell }}_{L}^{\beta } \gamma ^{\mu } q_{L}^{\,j} )\,, \nonumber \\ {{\mathcal {O}}}^{ij\alpha \beta }_{LR}&= ({\bar{q}}_{L}^{\,i} \gamma _{\mu } \ell _{L}^{\alpha }) ({\bar{e}}_{R}^{\beta } \gamma ^{\mu } d_{R}^{\,j} )\,, \nonumber \\ {{\mathcal {O}}}^{ij\alpha \beta }_{RR}&= ({\bar{d}}_{R}^{\,i} \gamma _{\mu } e_{R}^{\alpha }) ({\bar{e}}_{R}^{\beta } \gamma ^{\mu } d_{R}^{\,j}) \,. \end{aligned}$$

The normalization factor in the effective Lagrangian is $v=(\sqrt{2}\,G_F)^{-1/2} \approx 246$ GeV. We also introduce the effective scale $\Lambda _U = \sqrt{2} M_U/g_U$, such that

4 $$\begin{aligned} C^{33\tau \tau }_{LL} = \frac{ v^2 }{2 \Lambda _U^2 }\,. \end{aligned}$$

If we were interested only in $b\rightarrow c\tau {\bar{\nu }}$ transitions, we would have restricted our attention to the coefficients $C^{cb\tau \tau }_{LL(LR)}$.^1^ However, in order to also address the interplay with $b\rightarrow u\tau {\bar{\nu }}$ transitions and, most importantly, high-energy constraints, we need to analyze the relation among the $C^{cb\tau \tau }_{LL(LR)}$ and coefficients involving different quark flavors.

### Quark flavor structure

The flavor basis defined by $J_U^\mu$ can be considered the interaction basis for the LQ field. To address its relation to the mass-eigenstate basis of up (or down) quarks we need to write down and diagonalize the Yukawa couplings in this basis.

As in [3], we work under the assumption of an approximate $U(2)_f^3 = U(2)_Q \times U(2)_U \times U(2)_D$ symmetry acting on the light quark generations. In the limit of unbroken symmetry, the parameters $\epsilon _{q_k}$ in (2) should vanish and only third-generation quarks have non-zero Yukawa couplings. To describe a realistic spectrum, we proceed by introducing two sets of $U(2)_f^3$ breaking terms:

5 $$\begin{aligned}{} & {} {{\textbf{e}}}_q,~ {{\textbf{V}}}_{u},~ {{\textbf{V}}}_{d} ~\sim ~ \mathbf {2_Q}, \end{aligned}$$

6 $$\begin{aligned}{} & {} {\mathbf \Delta }_{u},~{\mathbf \Delta }_{d} ~\sim ~ {{\varvec{\bar{2}}}_{\textbf {U(D)}}}\times \mathbf {2_Q}, \end{aligned}$$

where ${{\textbf{e}}}_q$ denotes the vector ${{\textbf{e}}}^T_q = ( \epsilon _{q_1}, \epsilon _{q_2})$. The leading $\mathbf {2_Q}$ terms control the heavy$\, \rightarrow \,$light mixing in the left-handed sector, whereas the subleading ${{{\varvec{\bar{2}}}}_{\textbf {U(D)}}}\times \mathbf {2_Q}$ terms are responsible for the light Yukawa couplings.

The hypothesis of *minimal* $U(2)_f^3$ breaking, proposed in [41, 42] and employed in previous phenomenological analysis (see e.g. [3, 5, 40]), corresponds to the assumption of a single $\mathbf {2_Q}$ spurion, or the alignment of the three terms in (5) in $U(2)_Q$ space. Motivated by model-building considerations [22, 43] and recent data, we do not enforce this assumption in what follows. In addition to the minimal case, we will consider also the possibility of a (small) misalignment of the three leading $U(2)_Q$-breaking terms. We thus use the approximate $U(2)_f^3$ symmetry more as an organising principle to classify the flavor-violating couplings in the theory, rather than a strict ansatz on the underlying flavor structure.

Under these assumptions, the $3\times 3$ Yukawa couplings can be written as ($f=u,d$):

7 $$\begin{aligned} Y_f = y_{f_3} \left( \begin{array}{c|c} {\mathbf \Delta }_f &{} {{\textbf{V}}}_f \\ \hline 0 &{} 1 \end{array}\right) \,. \end{aligned}$$

Without loss of generality, the residual flavor symmetry allows us to choose a basis where both ${\mathbf \Delta }_u$ and ${\mathbf \Delta }_d$ are real. In this basis, the latter are diagonalised by a real orthogonal matrix,

8 $$\begin{aligned} \Delta _f = O_f \times \textrm{diag}\left( \frac{ y_{f_1} }{y_{f_3}} , \frac{ y_{f_2} }{y_{f_3}} \right) \,, \quad O_f = \left( \begin{array}{cc} c_f &{} s_f \\ -s_f &{} c_f \end{array}\right) \,, \end{aligned}$$

where $s_f =\sin \theta _f$ and $c_f =\cos \theta _f$, and ${{\textbf{V}}}_f$ are in general two complex vectors, ${{\textbf{V}}}^T_f = (V_{f_1}, V_{f_2})$.

The natural size of the different mixing terms can be deduced by the perturbative diagonalisation of $Y_u$ and $Y_d$. Introducing unitary matrices $L_f$, defined by

9 $$\begin{aligned} L_f Y_f Y_f^\dagger L_f^\dagger = \textrm{diag}( y_{f_1}, y_{f_2} , y_{f_3})\,, \end{aligned}$$

it follows that

10 $$\begin{aligned} L_f \approx \left( \begin{array}{c|c} O^T &{} 0 \\ \hline 0 &{} 1 \end{array}\right) \left( \begin{array}{c|c} 1 &{} -{{\textbf{V}}}_f^T \\ \hline {{\textbf{V}}}_f^\dagger &{} 1 \end{array}\right) \,. \end{aligned}$$

Since the elements of the Cabibbo, Kobayashi, Maskawa (CKM) matrix are given by $V_{ij} = (L_u L_d^\dagger )_{ij}$, we deduce

11 $$\begin{aligned} V_{u_2, d_2} = O(\lambda ^2)\,, \quad V_{u_1, d_1}= O(\lambda ^3)\,, \end{aligned}$$

where $$\lambda =|V_{us}| \approx 0.22$$, and

12 $$\begin{aligned} s_d-s_u = \lambda + O(\lambda ^3)\,. \end{aligned}$$

Assuming a common origin of the leading $U(2)_Q$-breaking terms, consistently with (11) it is natural to assume

13 $$\begin{aligned} \epsilon _{q_2} = O(\lambda ^2) \gg \epsilon _{q_1} ~. \end{aligned}$$

Everything discussed so far follows from the initial choice of symmetry breaking terms, as well as the requirement of reproducing the observed pattern of the quark Yukawa couplings. As we shall see, the non-observation of large deviations from the SM in ${\Delta F=2}$ transitions will impose further general constraints. This will allow us to pin down the precise relation between the Yukawa couplings and the LQ interaction basis.

#### Down-alignment of heavy$\,\rightarrow \,$light mixing.

In any realistic UV completion of the effective model considered here, there are also currents $J_q^\mu = {\overline{q}}^3_L\gamma ^\mu q^3_L$, associated to neutral mediators close in mass to the $U_1$ LQ. As discussed in [44], this is an unavoidable consequence of the closure of the algebra associated to $J^\mu _U$. In particular, this conclusion holds no matter if the $U_1$ is realized as a gauge boson or as a composite state. This fact implies that we also expect the effective interaction

14 $$\begin{aligned} \Delta {{\mathcal {L}}}^{4q} = O(1) \times \frac{1}{\Lambda _U^2} ({\overline{q}}^3_L\gamma ^\mu q^3_L)^2 \,. \end{aligned}$$

The latter can spoil the tight bounds on $B_{s(d)}$–${\bar{B}}_{s(d)}$ mixing unless the $V_{d_i}$ that control the off-diagonal entries of $L_d$ are about one order of magnitude smaller with respect to their natural size in Eq. (11).^2^ The smallness of these parameters makes them irrelevant for any other observable, so in the following we simply set ${{\textbf{V}}}_d = 0$. Under this assumption, the rotation matrices take the form

15 $$\begin{aligned} L_d \approx \left( \begin{array}{ccc} c_d &{} - s_d &{} 0 \\ s_d &{} c_d &{} 0 \\ 0 &{} 0&{} 1 \end{array}\right) \,, \qquad L_u = V \times L_d\,, \end{aligned}$$

and the only remaining free parameter in the Yukawa coupling is $s_u$ (or $s_d$), which controls the orientation of ${{\textbf{V}}}_u$ in $U(2)_Q$ space relative to the CKM vector $(V_{ub}, V_{cb})$^3^:

16 $$\begin{aligned} \frac{ V_{u_1} }{ V_{u_2}} = s_u + \frac{V_{ub}}{V_{cb}} + O(\lambda ^3) \end{aligned}$$

At this point it is convenient to re-write $J_U$ in the down-quark mass eigenstate basis by introducing the effective couplings $\beta _L^{ij}$ as in [15, 16]:

17 $$\begin{aligned} J_U^\mu =\frac{g_U}{ \sqrt{2} } \left[ \sum _{q=b,s,d} \beta _L^{q\tau } {\overline{q}}_L\gamma ^\mu \tau _L+\beta _R\,{\overline{d}}^3_R\gamma ^\mu e^3_R \right] \,. \end{aligned}$$

Using the expression of $L_d$ in Eq. (15) we get $\beta _L^{b\tau } = 1$ and

18 $$\begin{aligned} \beta _L^{s\tau }= & {} c_d \epsilon _{q_2} + s_d \epsilon _{q_1} = O(\lambda ^2) , \end{aligned}$$

19 $$\begin{aligned} \beta _L^{d\tau }= & {} c_d \epsilon _{q_1} - s_d \epsilon _{q_2} = O(\lambda ^3). \end{aligned}$$

Under the assumption of minimal $U(2)^3_f$ breaking, i.e. assuming the two ${{\textbf{2}}_Q}$ spurions ${{\textbf{e}}}_q$ and ${{\textbf{V}}}_u$ are aligned in $U(2)_Q$ space, it is easy to check that

20 $$\begin{aligned} \left. \frac{ \beta _L^{s\tau } }{ \beta _L^{d\tau } } \right| _{\text {minimal}~U(2)^3_f} = \frac{V_{td}^* }{ V_{ts}^*}\,. \end{aligned}$$

Therefore in the minimal case the value of the free parameter $s_u$ is irrelevant: it is absorbed into the definition of $\beta _L^{s\tau }$.

#### Non-minimal $U(2)_Q$ breaking with light-quark up alignment.

An interesting case worth considering from a model-building perspective is the limit $\epsilon _{q_1} \rightarrow 0$, or the limit where the LQ field does not couple to the first generation (in a generic basis where the light-family mixing is real). This limit necessarily implies a non-minimal $U(2)_Q$ breaking, or a misalignment between ${{\textbf{e}}}_q$ and ${{\textbf{V}}}_u$, as can be deduced by Eq. (16).^4^ As we discuss below, in this limit we are phenomenologically led to assume a real $\epsilon _{q_2}$ as well as approximate up alignment in the light-quark sector (i.e. $s_u\approx 0$), in order to evade the tight constraints from *K*–${\bar{K}}$ and *D*–${\bar{D}}$ mixing.

The ${\Delta F=2}$ constraints on the light-quark sector are more model dependent than those derived from ${\Delta B=2}$ transitions, since they depend on how the $U(2)_Q$ breaking is transferred from the LQ current to the neutral currents. If the latter preserve a $U(2)_Q$ invariant structure, then there is no constraint coming from the light-quark sector. However, it is not obvious how to justify this from a model-building point of view.

In the most realistic scenarios, $U(2)_Q$ is broken also in the neutral-current sector by terms proportional to appropriate insertions of ${{\textbf{e}}}_q$. In this case, and assuming ${{\textbf{V}}}_d = 0$, the severe constraint from CP-violation in ${\bar{K}}$–*K* mixing can be satisfied assuming a real ${{\textbf{e}}}_q$. However, this is not enough to simultaneously protect CP-violation in ${\bar{D}}$–*D* mixing. As pointed out recently in [43] (see also [33]), the latter forces us to choose $s_u \lesssim 0.1 \, \lambda$, i.e. an approximate up alignment in the light-quark sector.

In the phenomenological limit $s_u=0$ and ${{\textbf{V}}}_d = 0$, the light-quark fields in the interaction basis can be identified as

21 $$\begin{aligned} \left( \begin{array}{c} q_L^1 \\[2pt] q_L^2 \end{array} \right) = \left( \begin{array}{cc} V_{ud} &{} V_{us} \\[2pt] V_{cd} &{} V_{cs} \end{array} \right) \left( \begin{array}{c} d_L \\[2pt] s_L \end{array} \right) \approx \left( \begin{array}{c} u_L \\[2pt] c_L \end{array} \right) \,, \end{aligned}$$

while $q^3_L \equiv b_L$. The $\beta _L^{i\tau }$ become approximately diagonal in the up-quark mass basis and, setting $\epsilon _{q_1} \rightarrow 0$, we get

22 $$\begin{aligned} \beta _L^{c\tau } = \epsilon _{q_2}\,. \qquad \beta _L^{u\tau } = 0~. \end{aligned}$$

In the following we will investigate the relation between $b\rightarrow c$ and $b\rightarrow u$ transitions either assuming the minimal-breaking relation (20), or employing the ansatz (22).

### Charged currents in the mass-eigenstate basis

Following the notation of [16], we re-write the part of ${{\mathcal {L}}}^\textrm{LQ}_\textrm{EFT}$ relevant to $b\rightarrow c\tau {\bar{\nu }}$ transitions as

23 $$\begin{aligned} {{\mathcal {L}}}_{b\rightarrow c}&= - \frac{4 G_F}{\sqrt{2}} V_{cb} \bigg [ \Big ( 1 + {{\mathcal {C}}}_{LL}^{c} \Big ) ({\bar{c}}_L \gamma _\mu b_L) ({\bar{\tau }}_L \gamma ^\mu \nu _L) \nonumber \\&\quad - 2\,{{\mathcal {C}}}_{LR}^{c} \, ({\bar{c}}_L b_R) ({\bar{\tau }}_R\,\nu _L) \bigg ] \,, \end{aligned}$$

and similarly for $b\rightarrow u\tau {\bar{\nu }}$. The effective coefficients ${{\mathcal {C}}}^{c,u}_{LL(LR)}$ defined above are related to the coefficients in (3) by

24 $$\begin{aligned} {{\mathcal {C}}}_{LL(LR)}^c = \frac{ C_{LL(LR)}^{cb\tau \tau } }{V_{cb} }\,, \qquad {{\mathcal {C}}}_{LL(LR)}^u = \frac{ C_{LL(LR)}^{ub\tau \tau } }{V_{ub} }\,. \end{aligned}$$

Using the $\beta ^{ij}_{L}$ introduced in (17), we get

25 $$\begin{aligned} {{\mathcal {C}}}_{LL}^c= & {} C^{33\tau \tau }_{LL} \Bigg ( 1 + \sum _{i=s,d} \frac{V_{ci}}{ V_{cb} }\beta _L^{i\tau } \Bigg ) \equiv C^{33\tau \tau }_{LL}\Bigg ( 1+ \frac{\epsilon _q}{ |V_{cb}| }\Bigg ), \quad \nonumber \\ {{\mathcal {C}}}_{LR}^c= & {} \beta ^*_R \, {{\mathcal {C}}}_{LL}^c, \end{aligned}$$

where we defined the effective parameter $\epsilon _q$ to simplify the notation. Concerning the $b\rightarrow u$ coefficients, assuming the minimal-breaking relation (20) we get

26 $$\begin{aligned} {{\mathcal {C}}}_{LL(LR)}^u = {{\mathcal {C}}}_{LL(LR)}^c\,, \end{aligned}$$

whereas the non-minimal ansatz (22) leads to

27 $$\begin{aligned} {{\mathcal {C}}}_{LL(LR)}^u = \frac{ {{\mathcal {C}}}_{LL(LR)}^c }{1 + \epsilon _q/ |V_{cb}| }\,. \end{aligned}$$

## Observables

### Low-energy

The values of the effective couplings ${{\mathcal {C}}}^c_{LL}$ and ${{\mathcal {C}}}^c_{LR}$ can be fit at low energies using the experimental information on the LFU ratios $R_D$, $R_{D^*}$, and $R_{\Lambda _c}$. We have explicitly checked that other poorly measured observables, such as polarisation asymmetries in $b\rightarrow c\tau {\bar{\nu }}$ transitions or the loose bound on ${{\mathcal {B}}}(B^-_c \rightarrow \tau {\bar{\nu }})$ [45], do not currently provide additional constraints.^5^

The values of $R_D$ and $R_{D^*}$, recently measured by the LHCb collaboration, $R_D = 0.441 \pm 0.060_\textrm{stat} \pm 0.066_\textrm{syst}$, $R_{D^*}= 0.281 \pm 0.018_\textrm{stat} \pm 0.02 4_\textrm{syst}$, with correlation $\rho = -0.43$, shifts the world average of these ratios to [46]

28 $$\begin{aligned} R_{D^*}^\textrm{exp}= & {} 0.285 \pm 0.010_\textrm{stat} \pm 0.008_\textrm{syst}, \end{aligned}$$

29 $$\begin{aligned} R_{D}^\textrm{exp}= & {} 0.358 \pm 0.025_\textrm{stat} \pm 0.012_\textrm{syst}, \end{aligned}$$

with correlation $\rho = -0.29$. We fit these results within our model using the approximate numerical formulae reported in [16]:

30 $$\begin{aligned} \frac{R_D}{R_D^{\text {SM}}}&= |1+{{\mathcal {C}}}^c_{LL}|^2-3.00\, \textrm{Re}\left[ \left( 1+{{\mathcal {C}}}_{LL}^c\right) {{\mathcal {C}}}^{c\,*}_{LR}\right] \nonumber \\&\quad +4.12|{{\mathcal {C}}}^c_{LR}|^2\,, \end{aligned}$$

31 $$\begin{aligned} \frac{R_{D^*}}{R_{D^*}^{\text {SM}}}&= |1+{{\mathcal {C}}}^c_{LL}|^2-0.24\, \textrm{Re}\left[ \left( 1+{{\mathcal {C}}}_{LL}^c\right) {{\mathcal {C}}}^{c\,*}_{LR}\right] \nonumber \\&\quad +0.16| {{\mathcal {C}}}^c_{LR}|^2\,, \end{aligned}$$

where the Wilson coefficients are understood to be renomalized at the scale $\mu =m_b$. As reference values for the SM predictions we use the HFLAV averages [46]^6^:

32 $$\begin{aligned} R_{D}^{\text {SM}} = 0.298(4)\,,  R_{D^*}^{\text {SM}} = 0.254(5)\,. \end{aligned}$$

Concerning $R_{\Lambda _c}$, we use the approximate formula provided in [53], that in our notation reads

33 $$\begin{aligned} \frac{R_{\Lambda _c}}{R_{\Lambda _c}^\textrm{SM}} =&\,\, |1+{{\mathcal {C}}}^c_{LL}|^2-1.01\,\textrm{Re}\left[ {{\mathcal {C}}}^{c}_{LR}+{{\mathcal {C}}}_{LL}^c {{\mathcal {C}}}^{c\,*}_{LR}\right] \nonumber \\&+1.34|{{\mathcal {C}}}^c_{LR}|^2\,. \end{aligned}$$

As inputs we use the recent LHCb result, $R^\textrm{exp}_{\Lambda _c} = 0.242\pm 0.076$ [54], and the SM value $R_{\Lambda _c}^\textrm{SM} = 0.333(13)$ [53].

In the case of $b\rightarrow u \tau \nu$ transitions, the only relevant constraint at present is provided by ${\mathcal {B}}(B^-_u \rightarrow \tau \bar{\nu })$. Here the numerical expression reads [40]:

34 $$\begin{aligned} \frac{{\mathcal {B}}\left( B^{-}_u \rightarrow \tau \bar{\nu }\right) }{{\mathcal {B}}\left( B^{-}_u \rightarrow \tau \bar{\nu }\right) ^{\textrm{SM}}}=\left| 1+{{\mathcal {C}}}_{LL}^u-2\chi _u \, {{\mathcal {C}}}_{LR}^u\right| ^2 \, , \end{aligned}$$

where $\chi _u =m_{B^{+}}^2 /\left[ m_\tau \left( m_b+m_u\right) \right] \approx 3.75$. The data we use are ${\mathcal {B}}\left( B^{-}_u \rightarrow \tau \bar{\nu }\right) ^{\textrm{exp}}=1.09(24) \times 10^{-4}$ [55] and ${\mathcal {B}}\left( B^{-}_u \rightarrow \tau \bar{\nu }\right) ^{\textrm{SM}}=0.812(54) \times 10^{-4}$ [56].

**Fig. 1:**
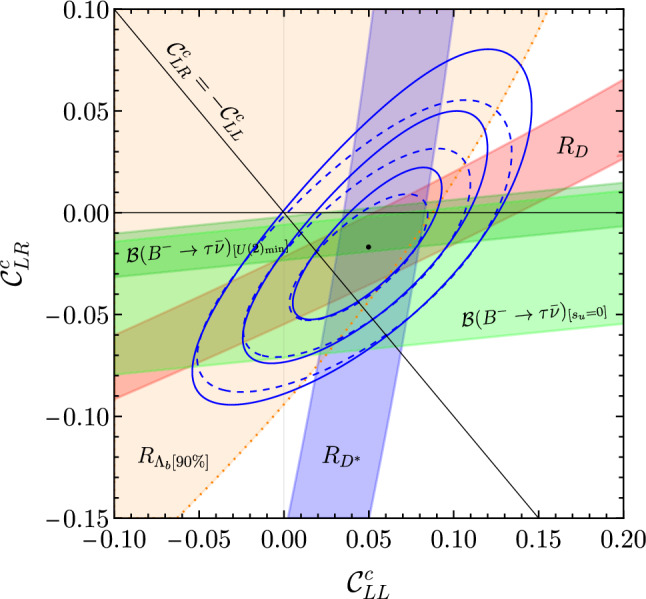
Determination of ${{\mathcal {C}}}^c_{LL}$ and ${{\mathcal {C}}}^c_{LR}$ from a $\chi ^{2}$-fit to low-energy observables. The Wilson coefficients, assumed to be real, are renormalized at the reference scale $\Lambda _\textrm{UV}=1$ TeV. The blue ellipses denote the 1, 2, and 3$\sigma$ contours fitting only $b\rightarrow c$ observables. The black dot indicates the best fit point of $(0.05,-0.02)$. The dotted lines are obtained including also ${\mathcal {B}}\left( B^{-}_u \rightarrow \tau \bar{\nu }\right)$ in the limit of up alignment. The $\Delta \chi ^2 = 1$ regions preferred by each observable are also indicated, except in the case of $R_{\Lambda _c}$ where we give the 90% CL region (due to the large error)

In Fig. 1 we report the best values of ${{\mathcal {C}}}^c_{LL}$ and ${{\mathcal {C}}}^c_{LR}$ as obtained from a $\chi ^{2}$-fit to the low-energy observables.^7^ The values reported in Fig. 1 correspond to the Wilson coefficients renormalized at a reference high-scale $\Lambda _\textrm{UV}=1$ TeV, which is the most appropriate scale to compare low- and high-energy observables. Taking into account only the QCD-induced running, we set ${{\mathcal {C}}}^c_{LL} (m_b) = {{\mathcal {C}}}^c_{LL} (\Lambda _\textrm{UV})$ and

35 $$\begin{aligned} {{\mathcal {C}}}^c_{LR} (m_b) = \eta _S \, {{\mathcal {C}}}^c_{LR} (\Lambda _\textrm{UV})\,, \qquad \eta _S\approx 1.6\,. \end{aligned}$$

The first point to notice is that the SM point $({{\mathcal {C}}}^c_{LL}={{\mathcal {C}}}^c_{LR}=0)$ is excluded at the $3\sigma$ level. The ${b\rightarrow c}$ observables favor a region compatible with both a pure left-handed interaction (${{\mathcal {C}}}^c_{LR}=0$) as well as the case with equal magnitude right-handed currents ${{\mathcal {C}}}^c_{LL}= -{{\mathcal {C}}}^c_{LR}$. In both cases, the pull of the $U_1$ LQ hypothesis with respect to the SM is $\Delta \chi ^2 = \chi ^2_\textrm{SM}-\chi ^2_\textrm{NP} \approx 11$, which is at the $3\sigma$ level. As first pointed out in [10], the case where ${{\mathcal {C}}}^c_{LL}= -{{\mathcal {C}}}^c_{LR}$ is a natural benchmark for a flavor non-universal gauge model, where both left- and right-handed third-family quarks and leptons are unified in fundamental representations of *SU*(4). As indicated by the dashed blue lines, the preferred region is essentially unchanged if ${\mathcal {B}}(B^- \rightarrow \tau \bar{\nu })$ is added under the hypothesis of non-minimal $U(2)_Q$ breaking and up-alignment. In either case, we find a best fit point of ${{\mathcal {C}}}^c_{LL}= 0.05$ and ${{\mathcal {C}}}^c_{LR} = -0.02$. On the other hand, the inclusion of ${\mathcal {B}}(B^- \rightarrow \tau \bar{\nu })$ under the hypothesis of minimal $U(2)_Q$ breaking (dark green band) disfavors sizable right-handed currents.

#### Loop-induced contribution to $b\rightarrow s\ell {\bar{\ell }}$

This analysis is focused on the leading couplings of the $U_1$ field to third-generation leptons. Hence, we do not discuss $b\rightarrow s\ell \ell$ transitions ($\ell =e,\mu$) in detail here. However, we recall that the operator ${{\mathcal {O}}}_{LL}^{sb\tau \tau }$ mixes via QED running [57] into operators with light leptons ($\tau {\bar{\tau }} \rightarrow \ell {\bar{\ell }}$ loop). This results into a lepton-universal contribution to the $b\rightarrow s\ell {\bar{\ell }}$ Wilson coefficient $C_9$ [58], defined according to standard conventions (see e.g. [59, 60]). We will estimate the size of this effect using the results of the fit in Fig. 1.

To this purpose, we note that besides the leading-log running from the high-energy matching scale (i.e. $M_U$) down to $m_b$, we should also include long distance (LD) contributions resulting from the one-loop matrix element of the semi-leptonic operator ${\mathcal {O}}^{sb\tau \tau }_{LL}$ [61]. Such contributions are analogous to the LD contributions from four-quark operators to the $b\rightarrow s\ell {\bar{\ell }}$ decay amplitude, which are present in the SM (see e.g. [62]). The only difference is that the charm loop is replaced by a tau-lepton loop. In full analogy to the factorizable part of the charm-loop contribution [62], also the (fully perturbative) LD tau-lepton contribution can be taken into account defining a $q^2$-dependent $C^\textrm{eff}_9(q^2)$, where $q^2=m_{\ell \ell }^2$. Considering also this effect, we find the following expression for the correction to $C^\textrm{eff}_9$ induced by the $U_1$:

36 $$\begin{aligned}{} & {} \Delta C_9^\textrm{eff} (q^2=0) = \frac{C^{sb\tau \tau }_{LL}}{V_{ts}^* V_{tb}} \frac{2}{3}\left[ \log {\left( \frac{M_U^2}{m^2_\tau }\right) }-1\right] , \nonumber \\{} & {} \quad = - \frac{ {{\mathcal {C}}}^c_{LL}}{1+|V_{ts}|/\epsilon _q} \frac{2}{3}\left[ \log {\left( \frac{M_U^2}{m^2_\tau }\right) }-1\right] .\quad \end{aligned}$$

The last expression follows from the relation between $C^{sb\tau \tau }_{LL}$ and ${{\mathcal {C}}}^c_{LL}$, which can be deduced from Sect. 2.1. For ${{\mathcal {C}}}^c_{LL}=0.05$ (best fit point in Fig. 1), $M_U=3$ TeV, and $\epsilon _q=2|V_{ts}|$, we get $\Delta C_9^\textrm{eff} (0) \approx - 0.3$. While not solving all $b\rightarrow s\ell {\bar{\ell }}$ anomalies, such a correction leads to a significant improvement in the description of $b\rightarrow s\ell {\bar{\ell }}$ data [16, 59, 60].

### High-energy

Collider observables are known to provide rich information on the parameter space of vector leptoquark models [31, 38, 44] explaining the *B*-meson anomalies, that is complementary to low-energy data [16, 63]. A variety of different underlying processes can be relevant at hadron colliders such as the LHC. The most important channels involving the $U_1$ leptoquark are:

- Pair production $$pp \rightarrow U_1^*U_1$$,
- Quark-gluon scattering $qg \rightarrow U_1 \ell$,
- Quark-lepton fusion $q\ell \rightarrow U_1$,
- Drell-Yan $$pp \rightarrow \ell {\bar{\ell }}$$.

The main decay channels in models where the leptoquark predominantly couples to third generation fermions are $U_1 \rightarrow b\tau ^+$ and $U_1 \rightarrow t \bar{\nu }_\tau$. In the case of interest where $g_U \gtrsim g_s$, the Drell-Yan production channel due to *t*-channel LQ exchange provides the most stringent constraints on the parameter space. Nevertheless, the other channels can still yield relevant information. For example, the searches for LQ pair production [64–66] set a lower bound on the $U_1$ mass of $M_{U}\gtrsim 1.7\,\text {TeV}$ [67, 68], which however only covers a small region of parameter space relevant for the explanation of the charged-current *B*-meson anomalies [16]. On the other hand, quark-gluon scattering [66, 69–71] and resonant production through quark-lepton fusion [72–76] will be important in case of a discovery, but they are not competitive at the moment.

Therefore, in the present analysis, we focus only on the non-resonant contributions of the $U_1$ vector LQ to Drell-Yan production. In particular, we are interested in the process $pp\rightarrow \tau {\bar{\tau }}$, with the main contribution due to $b\bar{b}\rightarrow \tau {\bar{\tau }}$, since we assume that the $U_1$ is predominantly coupled to third generation fermions. In such a scenario, the final state events are expected to contain an associated *b*-jet, due to gluon splitting $g \rightarrow b\bar{b}$ in the initial proton. We consider the CMS [36] and ATLAS [39] searches for the di-tau final state, based on the full LHC Run-II data sets. These searches provide results both in a *b*-tag channel, where an associated *b*-tagged jet is required in the final state, and in a *b*-veto channel, where the absence of any *b*-tagged jet is compulsory.

The contributions of the $U_1$ vector-leptoquark to Drell-Yan processes have recently been studied in Ref. [34] at next-to-leading order (NLO) in QCD. Notice that in any UV completion the $U_1$ leptoquark is expected to be accommodated by further degrees of freedom with masses in the ballpark of the $U_1$ mass, that will lead to additional collider signatures [9, 11, 16, 44]. These are, however, model dependent and thus not considered in the analysis at hand. Previous work investigating the connection of high-$p_T$ data with the low-energy observables for the *B*-meson anomalies can be found in Refs. [16, 63]. We extend these works by analysing the recent CMS di-tau search [36] in addition to the already previously investigated ATLAS search [39] for the same final state. Moreover, we use the results of Ref. [34] to extend the analysis incorporating NLO effects and to exploit the more constraining searches for di-tau final states in association with a *b*-jet.

**Fig. 2:**
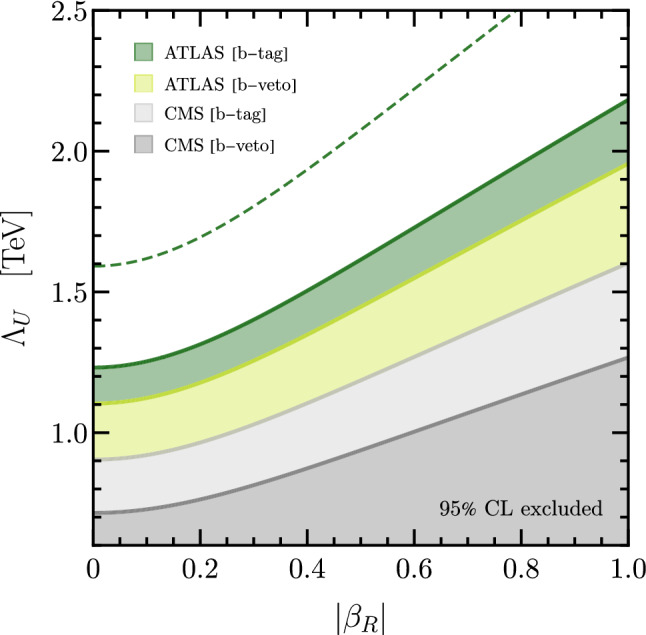
High-$p_T$ constraints on the $U_1$ model parameters $\beta _R$ and $\Lambda _U$ derived from the $pp\rightarrow \tau {\bar{\tau }}$ searches by CMS [36] (gray) and ATLAS [39] (green) in the *b*-tag and *b*-veto channels. The functional dependence is extracted using the HighPT package [63, 77] and rescaled to the results presented in Ref. [34]. The shaded regions correspond to the excluded parameter space at $95\%$ CL. The solid lines correspond to the constraints obtained using LHC run-II $(\sim 140\,\text {fb}^{-1})$ data, whereas the dashed line displays the projections for LHC’s high luminosity phase $(\sim 3\,\text {ab}^{-1})$ for the ATLAS *b*-tag search

For our present study we use the HighPT package [63, 77] to compute the $\chi ^2$ likelihood of the EFT Lagrangian in Eq. (3) for the *b*-veto channel of the ATLAS di-tau search [39]. We then rescale this result to match the NLO predictions derived in Ref. [34] for the $U_1$ leptoquark for the ATLAS [39] and CMS [36] searches in both *b*-tag and *b*-veto channels.^8^

Minimizing the rescaled $\chi ^2$ likelihoods with respect to the right-handed coupling $\beta _R$ and the effective scale $\Lambda _U$, we find the $95\%$ CL exclusion regions^9^ shown in Fig. 2. The ATLAS di-tau search [39], shown in green, provides stronger exclusion limits than the corresponding CMS search [36], displayed in gray. This can be understood by noticing that a slight excess of events is observed in the high-$p_T$ tail in the latter search, weakening the constraints derived from it. For both collaborations, the *b*-tag channels (dark green/light gray) yield more stringent constraints than the corresponding *b*-veto channels (light green/dark gray), as anticipated. As previously mentioned, this is because the signal comes dominantly from the process $b\bar{b}\rightarrow \tau {\bar{\tau }}$, where at least one bottom quark is likely to come from gluon splitting $(g\rightarrow b\bar{b})$ allowing to require an associated *b*-jet, which significantly reduces the background and thus yields stronger constraints. Furthermore, it is evident that the scenarios with large right-handed currents $\beta _R$ are tightly constrained by high-$p_T$ data.

**Fig. 3:**
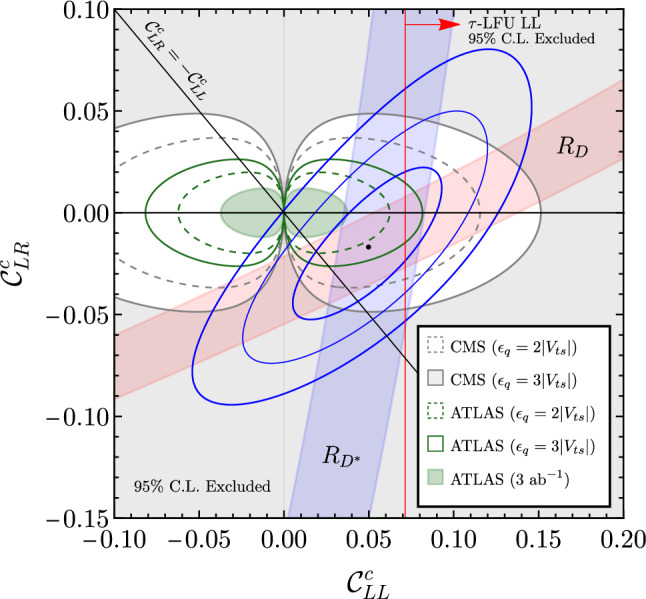
High-$p_T$ constraints superimposed on the low-energy fit. The red and blue bands represent the $\Delta \chi ^2 = 1$ regions preferred by $R_{D}$ and $R_{D^*}$. The blue lines correspond to the $1\sigma$, $2\sigma$, and $3\sigma$ contours of the combined low-energy fit including all $b\rightarrow c$ observables (dot = best fit point). The high-$p_T$ exclusion limits derived from the *b*-tag channel of the CMS [36] (ATLAS [39]) search are given by regions outside of the gray (green) lines. On the other hand, the region inside the innermost dotted curve is our projection for the allowed parameter space from high-$p_T$ searches (in absence of a signal) with a luminosity of $3\,\text {ab}^{-1}$. Finally, the region to the right of the red line is excluded by $\tau$-LFU tests assuming leading log running of $C^{33\tau \tau }_{LL}$. See text for more details

Next, we compare these high-$p_T$ results to the low-energy constraints derived in the previous section, by minimizing both likelihoods with respect to the Wilson coefficients ${\mathcal {C}}_{LL}^c$ and ${\mathcal {C}}_{LR}^c$, again evaluated at the reference high-scale $\Lambda _\textrm{UV}=1$ TeV. The resulting fit is shown in Fig. 3, where the red and blue bands represent the preferred $\Delta \chi ^2 = 1$ regions for the measurements of $R_{D}$ and $R_{D^*}$. The blue lines correspond to the $1\sigma$, $2\sigma$, and $3\sigma$ contours of the combined low-energy fit including all $b\rightarrow c$ observables, whereas the gray (green) lines indicate the $95\%$ CL exclusion contours for the CMS (ATLAS) di-tau search using the *b*-tag channel.^10^ The solid and dashed lines correspond to the constraints obtained assuming $\epsilon _{q}=3|V_{ts}|$ and $\epsilon _{q}=2|V_{ts}|$, respectively.

As can be seen, the high-energy constraints are already very close to the parameter region favored by low-energy data. To this purpose, it should be noted that scenarios with smaller $\epsilon _{q}$ are more constrained by high-$p_T$ as they require a lower scale $\Lambda _U$ to explain the charged-current anomalies (see Eq. (25)). On the other hand, values of $\epsilon _{q}$ larger than $3|V_{ts}|$ are both unnatural and highly disfavoured by $\Delta F=2$ constraints in UV complete models in the absence of fine-tuning.

Due to the excess of events currently observed by CMS, the corresponding limits are significantly weaker than those of ATLAS. If interpreted as a signal, the CMS excess (which is further supported by a dedicated *t*-channel analysis [37]) would favour the parameter region close to the CMS exclusion bounds in Fig. 3. Given the low-energy constraints, this would in turn prefer a scenario with sizable right-handed couplings. On the other hand, ATLAS data are more compatible with low-energy data in the region of a pure left-handed coupling (though right-handed couplings remain viable).

Overall, the plot in Fig. 3 shows that low- and high-energy data yield complementary constraints, and that a $U_1$ explanation of $R_{D^{(*)}}$ is compatible with present $pp\rightarrow \tau {\bar{\tau }}$ data. This plot also shows that future high-energy data will play an essential role in testing the $U_1$ explanation of charged-current *B* anomalies. To illustrate this point, we indicate the projection for an integrated luminosity of $3\,\text {ab}^{-1}$ by the shaded green central region in Fig. 3, which shows the potential of the high-luminosity phase of LHC assuming $\epsilon _{q}=2|V_{ts}|$. The projection was derived using the ATLAS *b*-tag search assuming that background uncertainties scale as the square-root of the luminosity. This projection shows that a large part of the relevant parameter space will be probed with the data sets expected from Run-III and the LHC high-luminosity phase.

For completeness, in Fig. 3 we also indicate the region disfavoured by LFU tests in $\tau$ decays [78]: the region to the right of the red line is excluded by the experimental determination of $(g_{\tau }^W / g^{W}_{\mu ,e})_{\ell ,\pi ,K}$ [46], using the leading-log (LL) running of $C^{33\tau \tau }_{LL} (1~\textrm{TeV})$ [78], and setting $\epsilon _{q}=3|V_{ts}|$ (most conservative choice). Due to their purely left-handed nature, $\tau$-LFU tests provide a strong constraint on the left-handed only hypothesis, potentially favouring scenarios with right-handed currents. However, this point comes with the caveat that additional contributions from new states in UV complete models can soften these bounds [79].

## Conclusions

In this paper we have analyzed the compatibility of the $U_1$ LQ explanation of the charged-current *B*-meson anomalies in light of new low- and high-energy data. To this purpose, we have first re-analysed in a bottom-up and, to large extent, model-independent approach the assumptions necessary to relate the $U_1$ couplings appearing in $b\rightarrow c\tau {\bar{\nu }}$, $b\rightarrow u\tau {\bar{\nu }}$, and $b {\bar{b}} \rightarrow \tau {\bar{\tau }}$ transitions.

Updating the fit to the low-energy data, we find that the region preferred by $b\rightarrow c$ observables is equally compatible with a purely left-handed interaction, as well as with a scenario with right-handed currents of equal magnitude. The latter option is quite interesting, given sizable right-handed currents are a distinctive signature of models where the $U_1$ is embedded in a flavor non-universal gauge group [10]. In both cases, the pull of the $U_1$ hypothesis is at the $3\sigma$ level. The present low-energy fit already highlights the role of $B_u \rightarrow \tau {\bar{\nu }}$ in pinning down the residual uncertainty on the flavor structure of the $U_1$ couplings. Indeed, this observable is expected to play an even more important role in the near future with the help of new data coming from Belle-II [80].

Next, we examined collider constraints on the model, focusing on the $pp\rightarrow \tau {\bar{\tau }}$ Drell-Yan production channel mediated by *t*-channel $U_1$ exchange that provides the most stringent bounds. By superimposing these limits on the parameter space preferred by the low-energy fit, we conclude that constraints coming from the high-energy $pp\rightarrow \tau {\bar{\tau }}$ process are already closing in on the low-energy parameter space preferred by the charged-current *B*-meson anomalies.

While low- and high-energy data are currently well compatible, a large fraction of the viable parameter space will be probed by the high-luminosity phase of the LHC. This is especially true in the case of equal magnitude left- and right-handed currents (${{\mathcal {C}}}^c_{LL}= -{{\mathcal {C}}}^c_{LR}$), which has become more viable with the updated low-energy data and will be probed at the 95% confidence level by the LHC. This will provide an exciting test of the well-motivated class of UV completions for the $U_1$ based on non-universal gauge groups, featuring quark-lepton unification for the third family at the TeV scale [10, 11, 20, 22].

## Note added

While this project was under completion, an independent phenomenological analysis of charged-current *B*-meson anomalies, including different leptoquark interpretations, has appeared [81]. Our results in Sect. 3 (low-energy fit) are compatible with those presented in [81].

## Figures and Tables

**Fig. 4:**
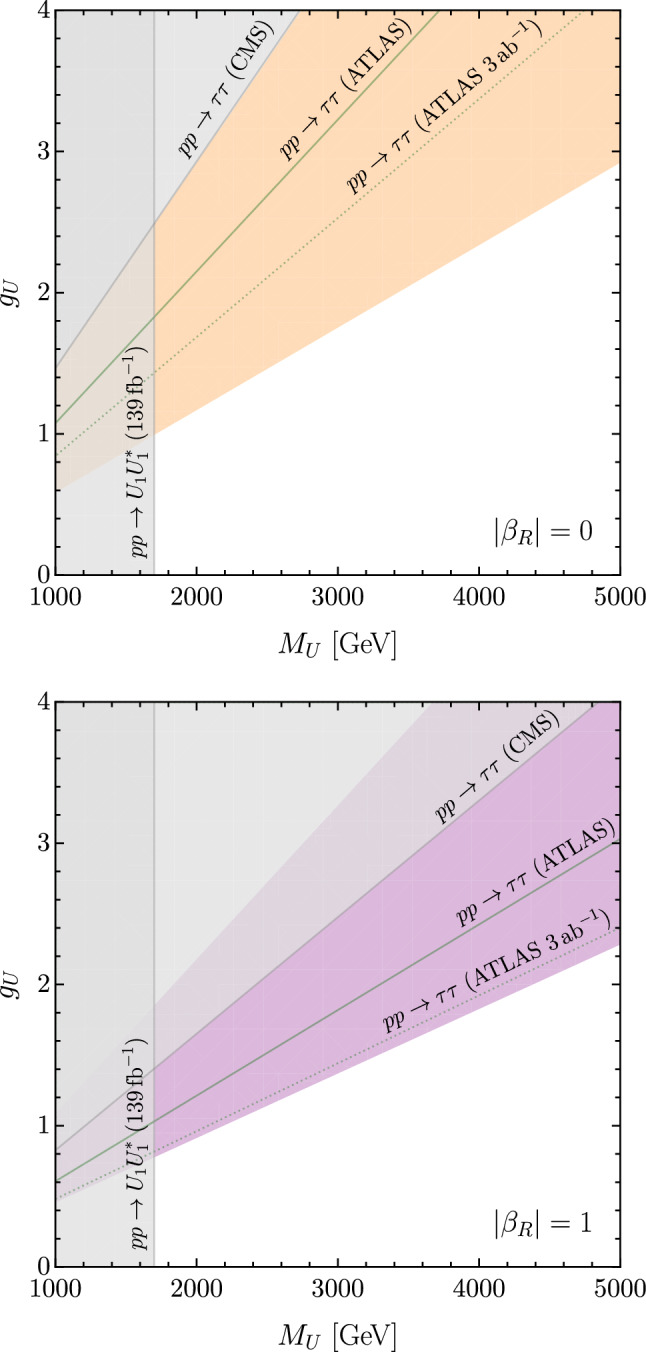
Preferred region at 90% CL from low-energy charged-current data for mass ($M_U$) and leading fermion coupling ($g_U$) of the $U_1$ LQ. Top: Purely left-handed case ($\beta _R=0$). Bottom: Pati-Salam-like case ($|\beta _R|=1$). The gray region and solid lines indicate constraints of present high-energy searches at 95% CL, while the dotted line gives the projected sensitivity at the HL-LHC with a luminosity of $3\,\textrm{ab}^{-1}$

**Fig. 5:**
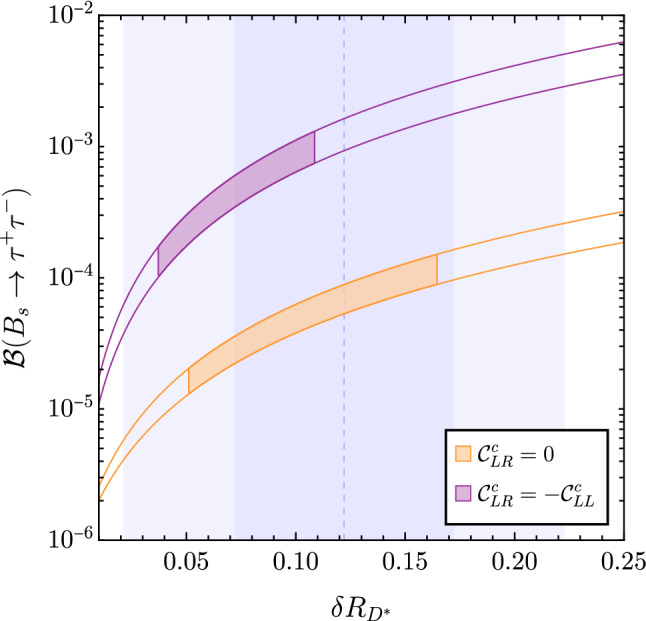
Predicted range for ${{\mathcal {B}}}(B_s \rightarrow \tau ^{+}\tau ^{-})$ as a function of $\delta R_{D^*}= R_{D^*}/R_{D^*}^{\text {SM}}-1$. The filled orange and purple colored regions correspond to the 90% CL preferred regions from the low-energy charged-current fit. The blue vertical bands denote the present $1\sigma$ and $2\sigma$ experimental ranges for $\delta R_{D^*}$

## Data Availability

This manuscript has no associated data or the data will not be deposited. [Authors’ comment: The data used in this paper are already published elsewhere.]

## Appendix A: Preferred regions for $U_1$ couplings

In view of future searches of $U_1$ signals in channels involving $\tau$ leptons, both at high and at low energies, we provide here a summary of the preferred parameter-space region resulting from the low-energy fit performed in this paper. We also report predictions for ${{\mathcal {B}}}(B_s \rightarrow \tau ^+\tau ^-)$, which can be considered the low-energy counterpart of $pp\rightarrow \tau {\bar{\tau }}$.

The effective interaction between the $U_1$ field and fermion currents involving the $\tau$ lepton is ${\mathcal {L}}_\textrm{int} = U_\mu J^{\mu }_U$, with $J^{\mu }_U$ defined as in (17). By convention, we set $\beta _L^{b\tau } = 1$. The parameter $\beta _R$, which characterises different UV completions of the effective interaction with right-handed fermions, should be treated as a free parameter. In order to define precise benchmarks, we consider two reference cases for $\beta _R$:

1. $|\beta _R| = 0$ (Purely left-handed case) Values preferred by low-energy data at 90% CL:
  A1 $$\begin{aligned} M_U/g_U&\in [0.69~\textrm{TeV},\, 1.71~\textrm{TeV}]\,, \end{aligned}$$
2. $|\beta _R| = 1$ (Pati-Salam-like LQ) Values preferred by low-energy data at 90% CL:
  A2 $$\begin{aligned} M_U/g_U&\in [0.92~\textrm{TeV},\, 2.19~\textrm{TeV}]\,. \end{aligned}$$

In Fig. 4 we show the present and future exclusion bounds in the $g_U$ vs. $M_U$ plane from high-energy searches, as well as the region preferred by the low-energy fit corresponding to (A1) and (A2).

As discussed in the main text, low-energy data on charged currents alone are not able to provide a stringent constraint on $\beta _{s\tau }$. However, the latter is constrained by $\Delta M_{B_s}$ under general assumptions about the UV completion. The range we consider motivated in view of future experimental searches is

A3 $$\begin{aligned} \beta _{s\tau } \in [0.06,\, 0.16]\,. \end{aligned}$$

The $g_U/M_U$ ranges reported in (A1) and (A2) are obtained under this assumption, and setting $\beta _{d\tau }= 0$.

As far as $B_s \rightarrow \tau ^{+}\tau ^{-}$ is concerned, the branching fraction can be decomposed as

A4 $$\begin{aligned} \frac{{\mathcal {B}}(B_s \rightarrow \tau ^{+}\tau ^{-})}{{\mathcal {B}}(B_s \rightarrow \tau ^{+}\tau ^{-})_\textrm{SM}} = \bigg |1+\frac{{{\mathcal {C}}}_\textrm{10,NP}^{s\tau }}{{{\mathcal {C}}}_\textrm{10,SM}}+\frac{\chi _{s\tau }{{\mathcal {C}}}_{P}^{s\tau }}{{{\mathcal {C}}}_\textrm{10,SM}}\bigg |^2&\nonumber \\ + \left( 1-\frac{4m_{\tau }^2}{m_{B_s}^2}\right) \bigg |\frac{\chi _{s\tau }{{\mathcal {C}}}_{S}^{s\tau }}{{{\mathcal {C}}}_\textrm{10,SM}}\bigg |^2&\,, \end{aligned}$$

where ${{\mathcal {C}}}_\textrm{10,SM} = -4.2$ and we have defined the chiral enhancement factor

A5 $$\begin{aligned} \chi _{s\tau } = \frac{m_{B_s}^2}{2m_{\tau }(m_b+m_s)} \,. \end{aligned}$$

In terms of the Wilson coefficients of ${{\mathcal {L}}}^\textrm{LQ}_\textrm{EFT}$, the other coefficients appearing in (A4) read

A6 $$\begin{aligned} {{\mathcal {C}}}_\textrm{10,NP}^{s\tau }&= \frac{2\pi }{\alpha V_{ts}^{*}V_{tb}} {{\mathcal {C}}}_{LL}^{sb\tau \tau } \,, \end{aligned}$$

A7 $$\begin{aligned} {{\mathcal {C}}}_{S}^{s\tau } = -{{\mathcal {C}}}_{P}^{s\tau }&= \frac{4\pi }{\alpha V_{ts}^{*}V_{tb}}{{\mathcal {C}}}_{LR}^{sb\tau \tau } \,. \end{aligned}$$

The model predictions for ${{\mathcal {B}}}(B_s \rightarrow \tau ^{+}\tau ^{-})$ corresponding to the two ranges in (A1) and (A2), as well as the flat range on $\beta _{s\tau }$ in (A3), are shown in Fig. 5.

## Footnotes

1 Here and in the rest of this section the up- or down-type flavor indices referred to $q_L^i$ indicate the corresponding $SU(2)_L$ doublet in a given (up- or down-type) mass eigenstate.

2 Precise bounds in 4321 gauge models have been discussed in [16].

3 Note that without loss of generality we can change the (overall) phase of the fields such that $V_{u_2}$ is real and set the CKM matrix to its standard phase convention.

4 Setting $\epsilon _{q_1}=0$ in a basis where the light-family mixing is real is equivalent to the statement that there is no non-trivial CP-violating phase between ${{\textbf{e}}}_q$ and $\Delta _{u,d}$. This prevents reproducing the physical phase in the CKM matrix using only these spurions. Indeed the (complex) relation (16) implies that the two components in ${{\textbf{V}}}_u$ have a different phase in the basis where ${\mathbf \Delta }_{u,d}$ are real.

5 Using the bound ${{\mathcal {B}}}(B^-_c \rightarrow \tau {\bar{\nu }}) \le 0.3$, derived in [45], we deduce $|{{\mathcal {C}}}^{c}_{LR}| \le 0.33$, which has no influence on the fit.

6 More details about the SM predictions of $R_D$ and $R_{D^*}$ and their uncertainties can be found in [47–52].

7 As can be seen from Eqs. (30–33), what matters for the low-energy fit in case of small Wilson coefficients is $\textrm{Re}({{\mathcal {C}}}_{LR}^c)$, so in the fit we take ${{\mathcal {C}}}_{LR}^c$ to be real for simplicity.

8 Reference [34] also provides results for the CMS search [37] for di-tau final states using angular observables. However, since such observables are currently not implemented in HighPT we refrain from rescaling our likelihood obtained for the total-transverse mass $m_{\tau {\bar{\tau }}}$ to this search.

9 The constraints presented in Fig. 2 are obtained assuming $\epsilon _{q}=2|V_{ts}|$, but only exhibit a very mild dependence on $\epsilon _{q_i}$.

10 Notice that the high-$p_T$ constraints are pinched at ${\mathcal {C}}_{LL}^c=0$ since this point corresponds to the limit $\beta _R\rightarrow \infty$ [see Eq. (25)].
